# Brazilian Journal of Anesthesiology: 75 years consolidating achievements

**DOI:** 10.1016/j.bjane.2026.844764

**Published:** 2026-05-12

**Authors:** Luiz Marciano Cangiani

**Affiliations:** Hospital da Fundação Centro Médico Campinas, Campinas, SP, Brazil

“*Here we open these welcoming pages hoping that the authors will satisfy our studious curiosity.”*

Renato Correa Ribeiro, 1951

The Brazilian Society of Anesthesiology (SBA), founded in 1948, published the first issue of its journal in 1951. Seventy-five years later, the Brazilian Journal of Anesthesiology (BJAN) remains one of the most meaningful expressions of Brazilian anesthesiology.

The inaugural editorial written by Ribeiro^1^ in 1951 was a mixture of joy, warning, faith, and hope. Actually, the emergence and continued growth of BJAN were only possible because of the philosophy implemented by the founders and absorbed by their followers, allowing its natural, yet methodical, development. Everything in parallel with the very development of Natural Sciences, in which concepts and behaviors, resulting from observation and experimentation, emerge based on empirical, scientific, philosophical, and theological knowledge.

Also in 1951, a copy of the journal was presented to the Editor-in-Chief of the journal Current Researches in Anesthesia and Analgesia, who wrote an editorial entitled "A New Journal is Born".^2^ This early international mention, later highlighted by Parsloe in an editorial, where he summarized much of the history of our journal, is particularly meaningful.^3^ It shows that, in addition to the internal objectives of the journal's publication, there was a desire to insert it into the international scenario.

The emergence of the journal, and its bimonthly publication schedule over the years, established a strong link between the Institution – the Brazilian Society of Anesthesiology – and its members.^4^ Stages were completed with dedication and hard work. Initially, the main objective was to have a journal that could disseminate knowledge and encourage national authors to publish their research, whether scientific or review articles, as well as presentations of clinical cases or anesthetic techniques, with the technical details of international publications. Annually, some selected articles, especially scientific ones, were published only in English in a special issue.

The great progress experienced over the years has been due to the inclusion of topics related to the philosophy of the scientific method in the scientific program of the Brazilian Congresses of Anesthesiology, held annually. The debates have not only consolidated the guidelines emanating from the most frequent and experienced authors, but also have encouraged younger, novice authors. This tradition continues to this day. Thus, our MDs, PhDs, and experts, of which there are many, join youth eager for knowledge, who will certainly become one of them in time. This is part of the SBA's objectives: "to bring together physicians interested in promoting the progress, improvement, and dissemination of anesthesiology, intensive care, pain management, palliative medicine, aerospace medicine, and resuscitation, and to establish standards for training in the specialty". As a result, most of the national articles originate from the 135 Anesthesiology Teaching and Training Centers accredited by the SBA, including those located in universities.

Until 2000, the journal was published in Portuguese, with abstracts in English and Spanish. In 2001, at the initiative of the Editorial Board, approved by the SBA Board of Directors, the journal became bilingual, with texts in Portuguese followed by an English translation. From then on, the number of citations of national papers increased.

In 2012, the publication began to present the articles in both Portuguese and English in full in the same issue. This led to a significant increase in submission from authors from other countries, requiring an expansion of the Editorial Board and the redefinition of goals and guidelines for the acceptance of articles.

In 2019, through the initiative of the Editorial Board and the essential support of the Board of Directors, the SBA decided that BJAN would be published only in English. The decision, implemented in 2020, made it easier to find articles in BJAN. The BJAN has definitively become more accessible to both national and international authors, definitively placing it within the international anesthesiology landscape.

Immersed in international literature, BJAN publishes articles from a variety of areas within anesthesiology, showing no preference for specific segments, as long as they adhere to scientific rigor and comply with author guidelines. Although the literature is a tangled web of a large puzzle, the excellent current databases allow for quick searches using well-applied keywords. BJAN, indexed in the institutions that offer such databases, has seen its impact factor gradually increase, reaching 1.9 in June 2025.

Up until the year 2000, the number of article submissions hovered around 250. Over the years, this number has grown, reaching 900 submissions in 2025. To comply with the analysis guidelines, flowchart, and schedule of the editorial process, the Editorial Board has grown significantly, currently comprising 1 Editor-in-Chief, 1 Co-editor, 28 Associate Editors, and 102 members of the Editorial Committee, specialists in the various areas of anesthesiology practice. Associate Editors and members of the Editorial Committee are appointed by the Editor-in-Chief and the Co-editor, subject to approval by SBA. The editorial work is carried out on a voluntary basis by all members of the BJAN Editorial Board. The workload is substantial, but the sense of fulfillment is even greater.

The Editor-in-Chief and Co-editor have always been elected by the SBA Assembly of Representatives for a three-year term, renewable for up to two more terms. However, since 2022, the Editor-in-Chief and Co-editor, with the same term length, are chosen by the SBA Board of Directors from a list of five names compiled by the Editorial Board. Since its launch, BJAN has had 12 Editors-in-Chief.

Brazilian anesthesiologists are very proud of BJAN, treating it with affection and respect. Thus, may future generations appreciate the great effort expended by many, and may they introduce the necessary modifications, with respect for their predecessors, in the constant pursuit of quality, seeking to consolidate achievements with courage and competence.

Therefore, the welcoming pages of BJAN remain wide open to the entire scientific community, hoping that the authors will continue to satisfy the curiosity of scholars.

## Data availability statement

No new data were created or analyzed in this study. Data sharing is not applicable to this article.

## Declaration of competing interest

The author declares no conflicts of interest.
